# Analysis of insulin resistance using the non-linear homeostatic model assessment index in overweight canines

**DOI:** 10.14202/vetworld.2022.1408-1412

**Published:** 2022-06-08

**Authors:** Franco Gonzlez-Villar, Francisco Pérez-Bravo

**Affiliations:** 1 Doctoral Program in Forestry, Agricultural and Veterinary Sciences, South Campus, University of Chile. Santa Rosa 11315, La Pintana, Santiago, CP 8820808, Chile; 2 Institute of Nutrition and Food Technology INTA, University of Chile, Santiago, Chile

**Keywords:** diabetes, glycemia, insulin resistance, overweight

## Abstract

**Background and Aim:**

Diabetes mellitus is a carbohydrate metabolism disorder produced mainly by a deficit in insulin production or insulin resistance. The homeostatic model assessment (HOMA) is a broad method for estimating insulin resistance and β-cell function. This study aimed to evaluate the stages of insulin resistance using non-linear HOMA index analysis in normoglycemic normal weight and obese canines.

**Materials and Methods:**

Insulin resistance was evaluated using the mathematical HOMA non-linear model in canines with different body and glycemic conditions. Forty canines were studied, including 20 normoglycemic normal weight canines and 20 normoglycemic obese canines. Chi-square statistical test was applied, in which the body condition and HOMA non-linear index were evaluated. The Spearman correlation test was conducted to evaluate the glycemic and insulin variables in both types of canines.

**Results:**

The Spearman correlation presented a correlation between increased blood glucose levels and insulin in obese canines, with a correlation of 0.79, while no significant changes in insulin were found in normal weight canines with different blood glucose levels, with a correlation of −0.11. The analysis of the non-linear HOMA index showed significant differences between non-linear HOMA insulin resistance in normal weight and obese canines, with a Chi-square statistic of 16.9424 and p = 0.000039. Canine with increased HOMA 2 showed higher levels of insulin with increasing blood glucose compared to those with normal HOMA 2.

**Conclusion:**

The HOMA 2 is a marker for evaluating increased insulin resistance in obese dogs and can be used to determine patients at risk for glycemic alterations.

## Introduction

Diabetes mellitus is a disorder in carbohydrate metabolism in which there is an increase in glucose levels at the blood level. Diabetes mellitus is mediated by environmental factors, such as high-fat diets and lack of exercise, and genetic factors. This rise in glycemia levels may be due to a failure to produce partial or absolute insulin and a loss of insensitivity to insulin by tissues [1]. Triggers for diabetes mellitus include genetic predisposition, infections generating states of insulin resistance in insulin receptors, increasing cortisol [2], insulin-resistant drugs (i.e., corticoids and progestogens), obesity, and pancreatitis. This interaction between genetic predisposition and insulin-resistant factors results in an irreversible loss of pancreatic b-cell function, along with a state of insulin resistance [3].

In veterinary medicine, it has been found that approximately 55% of canines and felines are overweight or obese, of which 45% are not investigated by owners [4]. The main problems that have been described associated with obesity are hypertension, hypercholesterolemia, increased levels of glycemia, insulin, and increased liver transaminases [5].

Obesity has been mainly attributed to secondary conditions, such as nutritional imbalance associated with high-fat diets, decreased physical activity, and endocrine problems such as hypothyroidism or Cushing’s disease [1]. However, several studies have established a possible etiology of a primary genetic origin of diabetes mellitus in canines, where genetic alterations similar to those found in human obesity have been described. Polymorphism alterations in the melanocortin receptors MCR4 and MCR3, the FTO gene that inhibits satiety, and in the peroxisome proliferator-activated receptor-gamma peroxisomal proliferation receptors are associated with obesity. However, their role in canine obesity has not yet been studied [6]. In the Beagle breed, two single-nucleotide polymorphisms have been found to affect the function of the MCR4 receptor, which is mainly associated with a lack of control in satiety. They are directly related to the generation of obesity due to increased food intake [7].

Obesity and its role in canine insulin resistance are controversial. Some authors have observed obesity-related metabolic dysfunction in canines and humans and described that 20% of obese canines have this dysfunction at the metabolic level [8]. When assessing the alteration involved in insulin resistance, as study conducted by Castro *et al*. [10] reported peripheral rather than hepatic resistance when canines were fed high-fat hypercaloric diets, both at the visceral and peripheral levels [9]. Despite this, some studies, mainly focused on the omental visceral adipose tissue in canines, have not found a correlation between this tissue and insulin resistance.

Australian studies assessing the possible factors involved in the absence of diabetes mellitus in obese canines found that canines with increased visceral adipose tissue show no decrease in adiponectin secretion [11]. Leptin is another cytokine involved in obesity and insulin resistance that also has been found in canines, which increases in obese patients compared to those of normal weight [12].

Some studies have attempted to develop methods to evaluate insulin resistance in canines by extrapolating them from human research. Among these methods, one of the most used is the measurement of insulin resistance through a mathematical model known as the homeostatic model assessment (HOMA). The HOMA-insulin resistance (HOMA-IR) has proved to be a robust tool for the surrogate assessment of insulin resistance; however, there is significant variability in the threshold HOMA-IR levels used to define IR by obesity in several populations [13].

There are two types of HOMA, the linear HOMA or HOMA 1 and the non-linear or HOMA 2. Some studies of obesity in canines have found an increase in linear HOMA in obese canines, considering a cutoff value of 2.5 [14], while the non-linear HOMA establishes cutoff values > 4.4; this method is helpful for patients with hyperglycemic states > 180 mg/dL [15].

The non-linear HOMA has been used to evaluate the effect of fish oil on adiponectin levels in canines [16]. The non-linear HOMA is a more modern model that evaluates variations in the liver and peripheral glucose resistance, showing, at the graph level, modifications in the curve of insulin secretion in response to a plasma glucose concentration > 180 mg/dL. The non-linear HOMA can also incorporate estimating pro-insulin secretion and consider renal glucose loss, facilitating its use in hyperglycemic individuals [12].

These models, despite being practical in clinical use, are inaccurate in determining the passage from insulin resistance to glucose intolerance, and as such, are mainly used in population-based medicine. In addition, the presence of pancreatic conditions frequently found in diabetic patients further affects the accuracy of this method [17]. The present study aims to evaluate the stages of insulin resistance by non-linear HOMA index analysis in normoglycemic normal weight and obese canines.

## Materials and Methods

### Ethical approval

The study was approved by Institutional Ethics Committee, University of Chile (CICUA) with approval no. 18194-VET-UCH.

### Study period and location

The study was conducted from March 2018 to December 2020 at the Clinical Hospital of the University of Chile and Medivet Hospital.

### Experimental design

Forty canines were studied, including 20 normoglycemic normal weight canines and 20 normoglycemic obese canines. The minimum sample size per group was set at 15 canines according to the calculation of means extrapolated from national incidence studies, using a confidence level 1-α of 95%, an expected loss ratio of 15%, and an accuracy of 3%.

Blood samples were collected by venipuncture of the cephalic vein and then placed in a tube without anticoagulant to measure serum glucose, measured immediately, and another with EDTA anticoagulant [18]. The sample with EDTA was used for insulin determination, centrifuged at 5000× g for 10 min at 21°C, before freezing at −80°C until analysis.

### Animals

The canines used were between 2 and 13 years old, without differentiation of sex due to the low number of male in the study (female-32 and male-8). All female canines in the study were neutered during the sample collection. Regarding breeds, the study comprised 15 mixed-breed canines, eight poodles, four beagles, four labradors, three golden retrievers, two cocker spaniels, one fox terrier, and three schnauzers.

The body condition of the canines was assessed using a 9-point morphometric body condition index, with an ideal body condition considered as a score of 5; establishing a condition 5, canines with palpable ribs, waist presence, and abdomen tucked up when viewed from the side; and obesity considered as a score of > 7 according to the World Small Animal Veterinary Association guidelines [19].

All canines underwent blood count and serum biochemistry tests, which showed that the levels of liver transaminases, creatinine, BUN, calcium, phosphorus, cholesterol, and albumin were all within range. The studied canines also underwent a routine clinical examination to evaluate the anamnesis, mucous membranes, lymph nodes, auscultation of the thoracic and abdominal, temperature, pressure, and skin [20]. Normal values of glycemia were considered between the range of 80–100 mg/ml. Glucose levels were measured using Mindray BS480 Valtek equipment, and normal insulin values were considered below 10 UmL.

### Analysis of the non-linear HOMA

The HOMA 2 or non-linear index was evaluated through the use of the HOMA calculator version 2.2.2 (Diabetes Trial Unit, University of Oxford), considering normal values < 4.4 (insulin values were approximately 2.9 uU/mL if insulin was lower than this range, and glycemia at 450 mg/dL if glucose was higher than this value) [15]. This index uses insulin and glucose for its calculation by incorporating an estimate of pro-insulin secretion into the model and renal glucose loss [13].

Insulin analysis was performed with the Perkin Elmer Wallac 1470–002 Wizard Gamma Counter DIAsource INS-IRMA, which has an intra-assay coefficient of variation of 0.29–2.93%, an inter-assay coefficient of variation of 1.12–3.16%, a standard internal coefficient of variation of < 2.27%, and a detection limit of 1.00 μI/mL.

The protocol for performing insulin IRMA involves the use of calibrators. First, 50 µL of the samples and control were placed in each tube before adding 50 μL of tracer to each tube [20].

### Statistical analysis

The variation in HOMA levels by breed and sex was analyzed using the analysis of variance test to identify the differences between the purebreds and mixed-breed canines. The Spearman correlation test was used to evaluate the glycemic and insulin variables in obese and normal weight canines.

Analysis was conducted using the χ^2^ test for categorical non-parametric variables, with 2 × 2 tables to assess the body condition and HOMA non-linear insulin resistance index to evaluate the association between variables. All confidence intervals are 95% level, a statistical significance of 5% was used, and data were processed in the statistical program StataCorp 2019 version 16.0 (StataCorp LLC, TX, USA).

## Results

Of the normal weight canines analyzed, 17 had non-linear HOMA values < 4.4 and three had non-linear HOMA values > 4.4. Among the obese canines, four had non-linear HOMA values < 4.4 and 16 had non-linear HOMA values > 4.4 (Table-1).

**Table 1:** Statistical analysis Chi-square between HOMA non-linear insulin resistance and weight.

Condition	HOMA < 4.4	HOMA > 4.4	Chi- square	p-value
Normoglycemic control	17	3	16.9424	0.000039
Normoglycemic obese	4	16		

There is a significant relationship between the variables HOMA non-linear insulin resistance and weight. HOMA=Homeostatic model assessment

**Figure-1:**
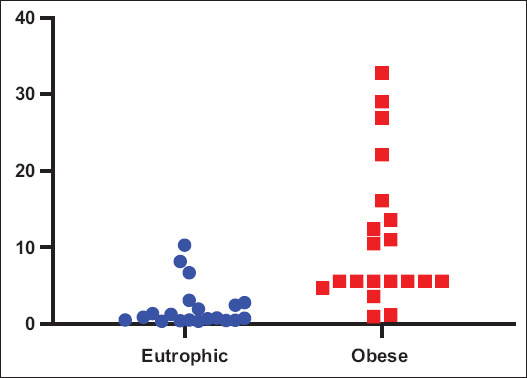
Analysis of the homeostatic model assessment 2 or non-linear between normal weight and obese, showing significantly higher levels in obese dogs than normal weight (p < 0.05).

The analysis of the non-linear HOMA index showed significant differences between non-linear HOMA insulin resistance in normal weight and obese canines (Figure-1), with a Chi-square statistic of 16.9424 and p = 0.000039 (Table-1).

**Figure-2:**
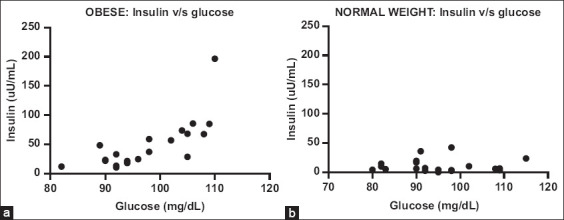
Insulin and glycemia in dogs with obese and normal weight. (a) Obese. As blood glucose increases, increases in insulin levels are observed. Spearman correlation 0.79 (b) normal weight. They do not observe changes in insulin as glucose increases. Spearman correlation −0.11.

The Spearman correlation presented a correlation between increased blood glucose levels and insulin in obese canines, with a significant correlation of 0.79 (glycemia mean: 10.5, SD: 5.9; insulin mean: 10.5, SD: 5.79). In contrast, no significant changes in insulin were found in normal weight canines with different blood glucose levels, with a correlation of −0.11 (glycemia mean: 10.5, SD: 5.89; insulin mean: 10.5, SD: 5.91) (Figure-2).

## Discussion

The study aimed to evaluate the stages of insulin resistance using non-linear HOMA index analysis in normoglycemic normal weight and obese canines. The obese canines diagnosed with insulin resistance with HOMA-2 > 4.4 showed glucose intolerance but not diabetes using a HOMA-2 out of 4.4 presented with various states of glucose intolerance but not diabetes. These results confirm that canines present different mechanisms for maintaining the glycemic state compared to humans [14]. HOMA-2 analysis to evaluate insulin resistance showed statistically significant differences across different body conditions. However, these results should be analyzed with caution. The HOMA is useful for evaluating insulin resistance at the epidemiological level in humans and has a high variability among patients at the clinical level [13]. Studies in veterinary medicine analyzing the changes in linear HOMA in normoglycemic and diabetic canines with different weights have shown no statistically significant differences between these groups [20].

The non-linear HOMA index of our study showed higher levels of HOMA index in obese normoglycemic canines compared to the normalweight control group. This finding shows that as the weight of canines increases, the HOMA index also increases due to an increase in insulin levels; this is consistent with the results of the previous studies on canine insulin resistance [15]. However, despite these increases, no canines with glycemic alterations were found during the follow-up period, which is consistent with canine canines who, despite presenting high glycemic states, maintain metabolic control with no increases in glycemia [11].

In young canines, there is the possibility of regenerating damaged islets, although this capacity decreases with age [21]. Canine diabetes occurs more frequently in females than in males; this higher prevalence in females could be explained by factors linked to generating insulin resistance in these canines, such as estrous cycles or pregnancy, given that at this stage, there is an increase in progesterone, a counter-regulating hormone of insulin that generates insulin resistance [22]. Progesterone also induces the expression of the growth hormone gene in the mammary gland of canines, which causes insulin resistance and, in some cases, diabetes mellitus [23]. Another factor proposed to account for the higher prevalence of canine diabetes in females is the decreased metabolism in sterilized females, which generates both visceral and peripheral obesity [1].

Genetic predisposition, infections, insulin-resistant drugs, obesity, pancreatitis, and various chronic diseases have been shown to induce insulin resistance [24]; however, the canines included in the present study were healthy with no associated pathology, as determined by blood tests.

A similar study conducted by Wallace *et al*. [13] had used linear HOMA and found differences in insulin resistance and diabetes associated with obesity. However, in our study, the canines show signs of insulin resistance and obesity, but not diabetes. Non-linear HOMA analysis may be a more helpful indicator than the evaluation of insulinemia alone because obese canines do not lose first-phase insulin secretion [11]. The non-linear HOMA measurement represents a promising indicator to continue studying canines.

## Conclusion

The HOMA 2 is a useful marker to evaluate increased insulin resistance in obese patients and can be used to determine those patients at risk for glycemic alterations. In the present study, no glycemic changes were found in canines with altered HOMA. In humans, the insulin deficiency status is expected to be above insulin resistance in patients with dysglycemia, this has not been clearly observed in canines demonstrating metabolic differences between humans and canines. Therefore, further studies are required, mainly focused on autoimmune disorders to complement the underlying mechanisms of dysglycemia in this species.

## Authors’ Contributions

FG: Study design, dog patient selection, conducted the experiment, and discussion of results. FPB: Study design, funding, discussion of results, and drafting of the manuscript. Both authors have read and approved the final manuscript.
